# Short- and Long-Term Effects of DBS on Gait in Parkinson's Disease

**DOI:** 10.3389/fneur.2021.688760

**Published:** 2021-10-08

**Authors:** Hana Brozova, Isabelle Barnaure, Evzen Ruzicka, Jan Stochl, Ron Alterman, Michele Tagliati

**Affiliations:** ^1^Department of Neurology and Centre of Clinical Neuroscience, First Faculty of Medicine, Charles University and General University Hospital in Prague, Prague, Czechia; ^2^Department of Neuroradiology, Kantonsspital Aarau, Aarau, Switzerland; ^3^Department of Kinanthropology, Charles University in Prague, Prague, Czechia; ^4^Department of Psychiatry, University of Cambridge, Cambridge, United Kingdom; ^5^Department of Neurosurgery, Beth Israel Deaconess Medical Center, Boston, MA, United States; ^6^Department of Neurology, Cedars-Sinai Medical Center, Los Angeles, CA, United States

**Keywords:** Parkinson's disease, DBS, long-term effect, gait, postural instability

## Abstract

The aim was to compare the short and long-term effects of subthalamic nucleus (STN) deep brain stimulation (DBS) on gait dysfunction and other cardinal symptoms of Parkinson's disease (PD). Two groups of patients were studied. The first group (short-term DBS, *n* = 8) included patients recently implanted with STN DBS (mean time since DBS 15.8 months, mean age 58.8 years, PD duration 13 years); the second group (long-term DBS, *n* = 10) included patients with at least 5 years of DBS therapy (mean time since DBS 67.6 months, mean age 61.7 years, PD duration 17.1 years). Both groups were examined using the Unified Parkinson's Disease Rating Scale (UPDRS) and Gait and Balance scale (GABS) during four stimulation/medication states (ON/OFF; OFF/OFF; OFF/ON; ON/ON). Data were analyzed using repeated measures ANOVA with time since implantation (years) between groups and medication or DBS effect (ON, OFF) within groups. In the short-term DBS group, stimulation improved all UPDRS subscores similar to dopaminergic medications. In particular, average gait improvement was over 40% (*p* = 0.01), as measured by the UPDRS item 29 and GABS II. In the long-term DBS group, stimulation consistently improved all clinical subscores with the exception of gait and postural instability. In these patients, the effect of levodopa on gait was partially preserved. Short-term improvement of gait abnormalities appears to significantly decline after 5 years of STN DBS in PD patients, while effectiveness for other symptoms remains stable. Progressive non-dopaminergic (non-DBS responsive) mechanisms or deleterious effects of high frequency STN stimulation on gait function may play a role.

## Introduction

Deep brain stimulation (DBS) is a successful treatment for advanced Parkinson's disease (PD) complicated by fluctuations of response to levodopa and/or levodopa-induced dyskinesias. Long-term (up to 11 years) benefits of subthalamic nucleus (STN) DBS have been reported for tremor, rigidity, bradykinesia, and motor fluctuations (1–6); however, the long-term effects on gait abnormalities, which are among the most disabling symptoms of advanced PD, are a matter of controversy (7). A large meta-analysis documented the short-term benefits (up to 2 years) of both STN and GPi stimulation on parkinsonian gait (8, 9), a finding that was confirmed using more detailed analyses of gait parameters (10–15) and balance control (14, 16–19).

Unfortunately, the short-term benefits of DBS on parkinsonian gait and postural abnormalities appear to decline over time (1, 3–6, 20–24). Gradual gait deterioration in PD patients after chronic DBS is usually attributed to the natural progression of the disease, but the cause may also be attributed to a decreasing beneficial effect or even a negative effect of long-term stimulation specifically on gait. Indeed, gait worsening is considered by some authors as a complication of chronic DBS therapy (14, 25).

The purpose of this study was to measure the short- and long-term effects of STN DBS on the cardinal symptoms of PD (i.e., tremor, bradykinesia, rigidity) in comparison to gait and postural abnormalities. We hypothesized that, if the deterioration of DBS benefits is due to the natural generalized progression of the disease, the response to DBS of all motor symptoms should show similar degrees of worsening over time. In other words, despite the predictable worsening of the OFF medication scores caused by disease progression, the positive effects of DBS should remain proportionally similar over time among various measures of motor function. In contrast, if the response to stimulation of only some symptoms deteriorates over time, then more complex explanations than simple disease progression might be required.

## Materials and Methods

### Study Subjects

We prospectively enrolled 18 consecutive PD patients attending the Movement Disorders Center. All of them previously underwent bilateral STN DBS implantation with the same surgeon (RA). Patients enrolled in the study were divided into two groups based on the duration of DBS treatment. A short-term group included eight patients (mean age 59, SD 4.6 years; disease duration 13, SD 4.6 years; duration of the disease at the time of stimulation 11, SD 5 years) with postoperative follow-up ranging between 1 and 2 years (mean 16, SD 7 months). A long-term group included 10 subjects (mean age 62, SD 10 years; disease duration 17, SD 5.1 years; duration of the disease at the time of stimulation 11.4 SD 5.4 years) who had received STN stimulation for ~5 years (mean 68, SD 14 months).

### Clinical Evaluations

The Unified Parkinson's Disease Rating Scale (UPDRS) and the Gait and Balance Scale (GABS) were used to assess symptom severity. GABS, which is designed to assess gait, freezing of gait, balance, and posture, consists of three parts: GABS I: historical information; GABS II: measurement of 14 gait and balance parameters and timed items; and GABS III: including time to cover 14 m walking at a preselected speed, time to walk as quickly as possible, and time to perform the stand-walk-sit test (SWS) (26). Examinations were evaluated and compared for the two study groups. One investigator (HB) performed all of the evaluations, utilizing videotaped patient examinations. Each patient was initially evaluated in the OFF medication state (i.e., at least 12 h following the last dose of levodopa) with the stimulators on (ON stim/OFF med). Stimulation was then discontinued for at least 30 min so that patients could be evaluated in the OFF stim/OFF med state. Subsequently, the patients took their usual morning medication dose and were examined a third time in the OFF stimulation/ON medication (OFF stim/ON med) state. Finally, patients were evaluated in the ON/ON condition 30–60 min after switching DBS back on to their individualized therapeutic settings.

The effects of stimulation were assessed by comparing the UPDRS-III and GABS scores in the ON stim/OFF med condition to those in the OFF/OFF condition. The ON medication conditions were also analyzed to test the effects of the dopaminergic medications. In addition to the total UPDRS-III and GABS scores, specific subscores for tremor, rigidity, bradykinesia, gait, and postural instability and gait disorders (PIGD) were evaluated independently.

The study was performed in accordance with the declaration of Helsinki and approved by the local institutional review board. Informed consent was obtained from each patient.

### Statistical Analysis

We used a repeated-measures analysis of variance (ANOVA) for intergroup comparisons, employing the number of years after DBS (1 and 5) as the intergroup fixed factor. The medication (ON, OFF) and stimulation status (ON, OFF) were intragroup fixed factors. The Tukey–Kramer multiple comparison *post-hoc* test was used to assess statistical significance. For an easy-to-survey expression of improvement in each of the symptoms with patients in the ON and OFF drug condition, we used rating percentage. Statistical significance level was set to 5%.

## Results

The average UPDRS III scores in the OFF/OFF state, a surrogate measure of the untreated severity of PD, were higher in the long-term DBS group (46 vs. 38 in the short-term DBS group) although this difference did not reach statistical significance (Table 1).

**Table 1:** The mean values of UPDRS III and GABS II in all four states (stim/med) in patients with short and long-term stimulation (*P*-value).

**Items**		**Tremor**	**Rigidity**	**Brady**	**Gait**	**PIGD**	**UPDRS III**.	**GABS II**.
		**0–20**	**0–20**	**0–32**	**0–4**	**0–16**	**0–108**	**0–67**
**Patients**								
OFF med	Short-term DBS OFF stim	4.6	8.9	12.5	1.5	4.6	38.0	16.3
	Short-term DBS ON stim	0.9	6.4	8.9	0.9	3.3	23.8	9.3
	*P*-value On stim vs. Off stim	0.05	0.01	0.04	0.01	<0.01	0.002	<0.001
	Long-term DBS OFF stim	3.2	8.7	19.3	2.0	6.5	46.0	25.1
	Long-term DBS ON stim	0.3	4.7	11.6	1.8	5.7	28.3	21.3
	*P*-value ON stim vs. OFF stim	0.003	<0.001	<0.001	0.34	0.12	<0.001	0.03
ON med	Short-term DBS OFF stim	2.1	5.0	8.4	0.9	2.4	22.4	7.0
	Short-term DBS ON stim	0.3	2.4	4.8	0.3	1.0	10.8	4.6
	*P*-value ON stim vs. OFF stim	0.08	<0.01	<0.01	<0.05	0.01	<0.01	<0.05
	Long-term DBS OFF stim	0.9	4.7	12.1	1.3	5.1	29.2	17.8
	Long-term DBS ON stim	0.1	1.8	6.8	1.3	3.9	16.0	15.1
	*P*-value ON stim vs. OFF stim	0.12	0.003	0.002	1.0	<0.01	0.002	0.16

*PD, Parkinson's disease; UPDRS, Unified Parkinson's Disease Rating Scale; DBS, Deep Brain Stimulation; GABS, Gait and Balance Scale; PIGD, Postural instability and gait disorders*.

In the ON/OFF state, short-term DBS subjects showed significant improvement in response to stimulation in all of the measures examined with the exception of two walking tests: usual speed walking and the SWS test (Tables 1, 2). Long-term DBS subjects also showed significant improvement in the total UPDRS III score, tremor, rigidity, bradykinesia, and GABS II; however, gait, PIGD, and all the timed walking tests failed to improve with stimulation (Tables 1, 2).

**Table 2:** Timed gait tasks (GABS III) in all four states in PD patients with short- and long-term stimulation (*P*-value).

**Parameters of gait (path 14 m)**		**Time of walking at usual speed (s)**	**Time of walking as fast is possible (s)**	**SWS test (s)**
**Patients**				
OFF med	Short-term DBS OFF stim	20.8	16.1	18.4
	Short-term DBS ON stim	18.3	14.1	18.4
	*P*-value ON stim vs. OFF stim	0.15	<0.001	0.99
	Long-term DBS OFF stim	27.9	24.3	39.0
	Long-term DBS ON stim	25.7	22.9	29.8
	*P*-value ON stim vs. OFF stim	0.10	0.18	0.14
ON med	Short-term DBS OFF stim	19.5	15.1	17.5
	Short-term DBS ON stim	17.1	14.2	17.1
	*P*-value ON stim vs. OFF stim	<0.01	0.05	0.42
	Long-term DBS OFF stim	22.2	19.5	24.6
	Long-term DBS ON stim	23.2	19.8	28.9
	*P*-value ON stim vs. OFF stim	0.48	0.71	0.46

*PD, Parkinson's disease; GABS, Gait and Balance Scale; SWS, Stand-walk-sit test; DBS, Deep Brain Stimulation*.

In the ON/ON state, short-term DBS subjects showed further significant improvement in response to stimulation in all measured parameters except for the SWS test. The long-term DBS group improved significantly in the total UPDRS III score, rigidity, and bradykinesia. No significant improvement was noted in tremor, PIGD, or GABS parameters. The UPDRS III gait subscore remained unaffected, and the outcome of all quantitative walking tests showed worsening although the differences were not significant. The difference in percentage improvement between the short- and long-term DBS groups in the ON/ON vs. OFF stim/ON med state was even more striking. The PIGD score improved by 29.7% in the short-term DBS group as compared with 12.3% in the long-term DBS group. Similarly, GABS objective parameters improved 43% in the short-term DBS group as compared with 15% in the long-term DBS group; for gait rating, the difference was 70 vs. 0% (Table 3).

**Table 3:** Percentage change (decrease) of UPDRS III and GABS II scores after turning stimulation ON in PD patients with short- and long-term stimulation.

**Items**		**Tremor**	**Rigidity**	**Bradykinesia**	**Gait**	**PIGD**	**UPDRS III**	**GABS II**
**Patients**								
OFF med	Short-term DBS	81.0	28.2	29.0	41.7	29.7	37.5	43.1
	Long-term DBS	90.6	46.0	39.9	10.0	12.3	38.5	15.1
ON med	Short-term DBS	86.5	52.5	43.6	70.0	58.8	52.1	33.9
	Long-term DBS	89.9	61.7	43.8	0	23.5	45.2	15.2

*UPDRS, Unified Parkinson's Disease Rating Scale; GABS, Gait and Balance Scale; PD, Parkinson's disease; DBS, Deep Brain Stimulation; PIGD, Postural instability and gait disorders*.

**Figure 1:**
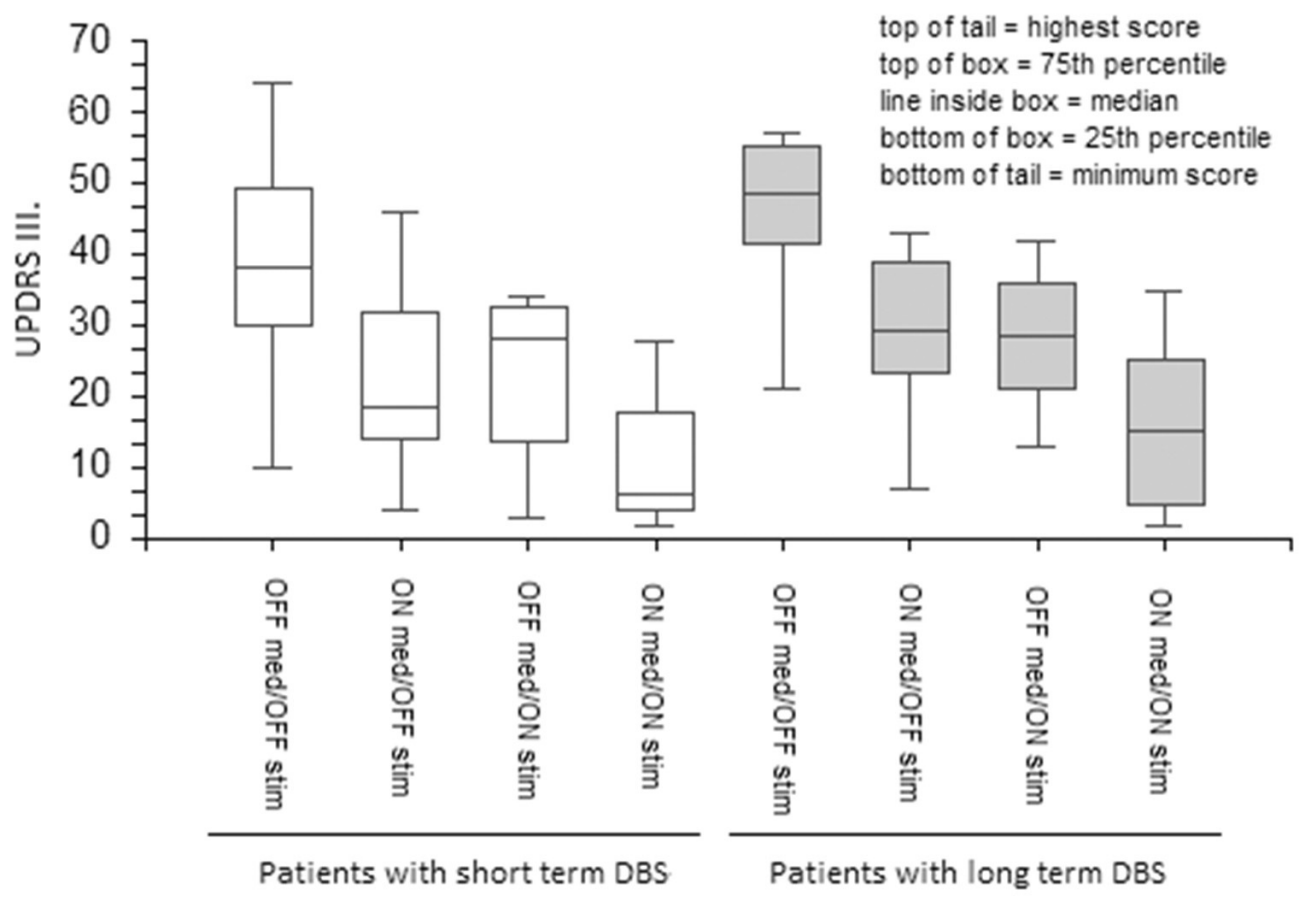
The effect of medication and stimulation on UPDRS III in patients with short- and long-term DBS. UPDRS, The Unified Parkinson's Disease Rating Scale; DBS, Deep Brain Stimulation.

**Figure 2:**
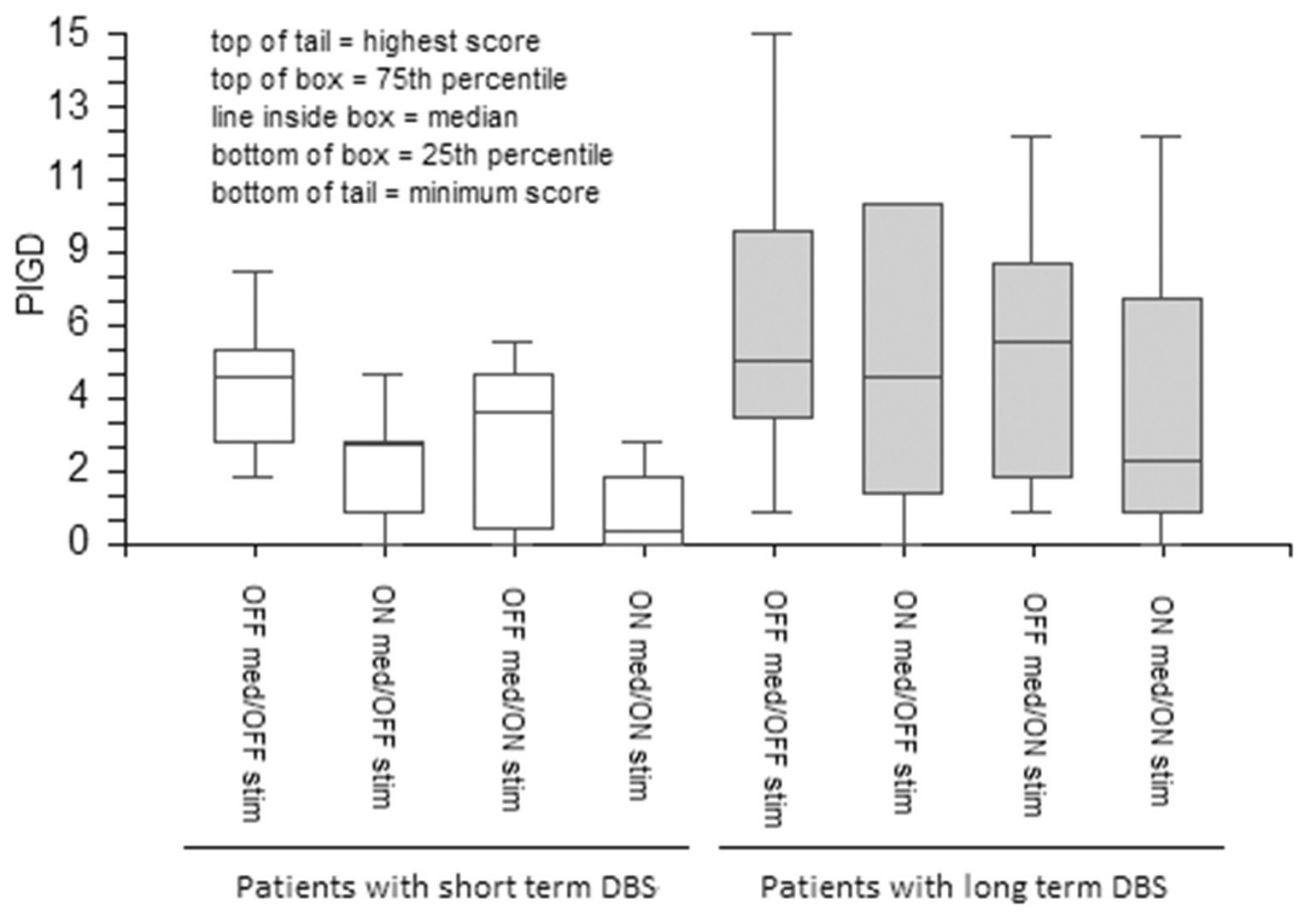
The effect of medication and stimulation on PIGD (items 27–30 of UPDRS) in patients with short- and long-term DBS. PIGD, Postural instability and gait disorders; PD, Parkinson's disease; UPDRS, The Unified Parkinson's Disease Rating Scale; DBS, Deep Brain Stimulation; GABS, Gait and Balance Scale.

DBS and medications equally ameliorated UPDRS III total scores in the short- and long-term DBS patients (Figure 1). However, although both stimulation and medication had positive effects on the PIGD subscore in the short-term DBS group, only medication improved PIGD in long-term DBS patients by a minimal margin (Figure 2).

## Discussion

The results of this study confirm that short-term stimulation (1–2 years) has a positive effect on all PD symptoms, including balance and gait dysfunction. However, we observed a significant decline of the beneficial effects of STN DBS in patients treated for 5 years or more. In particular, gait and postural disability appeared to specifically deteriorate, and DBS effects on the other cardinal symptoms of PD remained robust. The effects of dopaminergic medications were relatively preserved in patients with long-term STN DBS, albeit to a lesser degree as compared with those observed in the short-term DBS group.

The observation of a decline of DBS efficacy on specific features of PD over time is not new and has been generally attributed to the unavoidable progression of axial symptoms as part of the natural course of PD (1, 3, 4, 20, 21). To test whether the declining benefits of DBS can be purely attributed to the progression of the disease, we compared DBS responses to the OFF condition at the follow-up visit as opposed to presurgical or baseline OFF. We hypothesized that, despite the predictable worsening of the OFF medication scores caused by the progression of the disease, DBS improvement should have been percentage-wise similar over time. As expected, we found that the UPDRS III score in the OFF/OFF condition was ~20% higher in the long-term DBS group, which included chronically stimulated patients with longer disease duration. Supporting the hypothesis of a consistent DBS effect over time, both groups showed a 38% average UPDRS III score improvement after DBS was switched ON. However, the percentage improvement of gait and balance functions (UPDRS items 27–31) dropped from 43% in short-term stimulation patients to 10% in their chronic DBS counterparts. In addition, no gait improvement was found in chronically stimulated patients in the ON/ON state. These results were confirmed using more sensitive gait and balance tests, providing further support to our analysis. Interestingly, UPDRS item 29 best correlated with the timed tests measuring walking as quickly as possible, and SWS and timed walking at usual speed did not. The difference could lie in the fact that the SWS test does not evaluate only gait and test of walking by usual speed tends to be slower regardless of the ability to walk faster in a given time. In conclusion, the decline of the DBS effect observed in PD patients with chronic DBS may not be only attributed to a natural progression of PD symptoms. A declining ability of DBS to improve axial symptoms, including gait and balance difficulties, seems to also play a specific role (22). This could be due to either the progressive “intrusion” of non-dopaminergic mechanisms (not responsive to DBS) in the PIGD pathogenesis (27) or a progressively deleterious effect of high-frequency stimulation on gait function. The residual, milder improvement observed with levodopa would support the first explanation. On the other hand, the reported benefit of switching stimulation to lower frequencies after 5 years of STN DBS (13) supports, at least in part, the second hypothesis. Moreover, a review of the low-frequency effect DBS on axial signs assumes that low frequency might be clinically useful mainly when it lessens the detrimental effects of high-frequency stimulation (28). In addition, DBS and levodopa might have a different synergic effect on (29) cardinal symptoms in contrast to gait disorders.

The main limitation of this study is the small number of patients who were not followed prospectively. Although the groups were comparable in age, time of disease duration, and duration of the disease at the time of stimulation, there might be an impact of interpatient variability on observed results. Further prospective studies with a more detailed analysis of gait parameters in larger cohorts of chronically stimulated patients are needed to clarify this crucial issue.

## Data Availability Statement

The raw data supporting the conclusions of this article will be made available by the authors, without undue reservation.

## Ethics Statement

The studies involving human participants were reviewed and approved by Mount Sinai School of Medicine Institutional Review Board. The patients/participants provided their written informed consent to participate in this study.

## Author Contributions

HB: study design, methodology, data curation, formal analysis, investigation, project administration, and writing—original draft. IB: data curation, investigation, and writing—review and editing. ER: conceptualization, supervision, and writing—review and editing. JS: data curation, formal analysis, and writing—review and editing. RA: data curation and writing—review and editing. MT: conceptualization, methodology, supervision, and writing—review and editing. All authors contributed to the article and approved the submitted version.

## Funding

This work was supported by Ministry of Health of the Czech Republic, Grant No. 17-32318A, recipient HB, http://www.azvcr.cz/databaze-projektu/ves-2017. IB's work was supported by a grant for doctoral candidate's of the German Academic Exchange Service (DAAD). The funders had no role in study design, data collection and analysis, decision to publish, or preparation of the manuscript.

## Conflict of Interest

The authors declare that the research was conducted in the absence of any commercial or financial relationships that could be construed as a potential conflict of interest.

## Publisher's Note

All claims expressed in this article are solely those of the authors and do not necessarily represent those of their affiliated organizations, or those of the publisher, the editors and the reviewers. Any product that may be evaluated in this article, or claim that may be made by its manufacturer, is not guaranteed or endorsed by the publisher.
